# Sensor Fabrication Method for *in Situ* Temperature and Humidity Monitoring of Light Emitting Diodes

**DOI:** 10.3390/s100403363

**Published:** 2010-04-07

**Authors:** Chi-Yuan Lee, Ay Su, Yin-Chieh Liu, Pin-Cheng Chan, Chia-Hung Lin

**Affiliations:** Department of Mechanical Engineering, Yuan Ze Fuel Cell Center, Yuan Ze University, Taoyuan, Taiwan; E-Mails: meaysu@saturn.yzu.edu.tw (A.S.); M77YCLIU@saturn.yzu.edu.tw (Y.C.L.); s975132@mail.yzu.edu.tw (P.C.C.); s975009@mail.yzu.edu.tw (C.H.L.)

**Keywords:** LED, MEMS, flexible micro temperature, humidity sensors

## Abstract

In this work micro temperature and humidity sensors are fabricated to measure the junction temperature and humidity of light emitting diodes (LED). The junction temperature is frequently measured using thermal resistance measurement technology. The weakness of this method is that the timing of data capture is not regulated by any standard. This investigation develops a device that can stably and continually measure temperature and humidity. The device is light-weight and can monitor junction temperature and humidity in real time. Using micro-electro-mechanical systems (MEMS), this study minimizes the size of the micro temperature and humidity sensors, which are constructed on a stainless steel foil substrate (40 μm-thick SS-304). The micro temperature and humidity sensors can be fixed between the LED chip and frame. The sensitivities of the micro temperature and humidity sensors are 0.06 ± 0.005 (Ω/°C) and 0.033 pF/%RH, respectively.

## 1. Introduction

Light emitting diodes (LED), which are environmentally friendly, consume little power and have a long lifetime, have caused a revolution in illumination in the 21st century. They are favored because of their high reliability, low power consumption, and little-to-no-maintenance requirements. They have been adopted for a long time in various devices and computers as visual indicators of signal integrity and operational status. An LED is a solid-state semiconductor device that directly converts electrical energy into light. It is a strong candidate for the next generation of general illumination applications. Following further improvements, LEDs have recently been identified as illumination devices. High-power, high-brightness light emitting diodes are being used in an increasing number of lighting applications because of their excellent color saturation and long lifetimes. Preventing them from overheating is challenging task for thermal designers. The thermal management of packages depends on external cooling mechanisms, heat dissipation, and thermal interfaces. Under a fixed cooling condition, the rate of increase of the junction temperature of an LED increases with thermal resistance, reducing the luminescent efficiency declines. Therefore, the effective thermal design of LED packages with low thermal resistance is essential to improving the performance of LEDs [1,2]. The temperature of the junction affects LED performance in a range of ways. The center wavelength, spectrum, power of the light output and reliability of the diode depend directly on the temperature of the junction. The junction temperature in an LED cannot be measured using presently available instruments. Cheng [3] estimated that the junction temperature of an ion-implanted LED, determined using a forward voltage method, is significantly lower than that of a conventional LED. Senawiratne [4] measured thermal resistance to determine the junction temperature of a light emitting diode, as the driving current was increased from 10 to 250 mA.

As well as temperature, humidity factor significantly influences LED packaging. Moisture causes delaminating in electronic packages. LED packages are molded using polymeric materials. Such moisture diffuses into the package, which therefore swells. The package swells after absorbing moisture. Furthermore, the mismatching coefficients of moisture expansion (CMEs) induce hydro-mechanical stress in the package [5]. Tan [6] investigated GaN-based packaged white LEDs using non-destructive failure analytical tools and accelerated humidity testing, determination of mixed statistical distribution, spectral analysis and parametric extraction. In particular, despite the fact that all packaged integrated circuits undergo humidity testing, few investigations have explored the effects of humidity on packaged LEDs. Additionally, packaged LEDs operate in moist environments in numerous applications. In this work, flexible micro temperature and humidity sensors were employed to monitor in-situ the temperature and humidity of a light emitting diode. The sensors were fabricated on a stainless steel foil substrate (SS-304 with a thickness of 40 μm) by micro-electro-mechanical systems (MEMS). The sensors that were fabricated using this technique were: (1) small, (2) highly sensitive, (3) flexible but with precise measurement positions, (4) able to be mass produced, and (5) multi-functional.

## 2. Methodology

### 2.1. Theory of Micro Temperature Sensor

**Figure 1.**
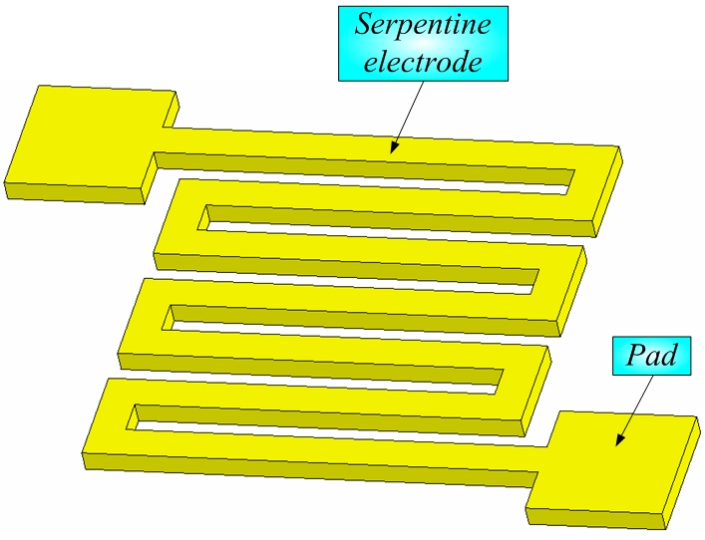
Structure of micro temperature sensor.

In this study, the micro temperature sensor was a resistance temperature detector (RTD). Among the various advantages of an array of thin film RTD sensors include a small volume, high accuracy, short response time, and mass production capacity. As Pt and Au are the conventionally adopted sensing materials in temperature sensors, the former is expensive while the latter has a higher conductivity and flexibility. Therefore, in this work, Au is used in the device [7]. As the environmental temperature increases, the resistance of the RTD also increases, because a metal conductor has a positive temperature coefficient (PTC). Figure 1 depicts the structure of the micro temperature sensor.

When the temperature of the RTD varies linearly, the relationship between the measured resistance and the change in temperature can be expressed as:

(1) $$R_{t}=R_{i}\;(1+\alpha_{T}\;T)$$

where *R_t_* represents the resistance at *t* °C; *R_i_* is the resistance at *i* °C, and α*_T_* is the sensitivity (1/°C).

Equation (1) can be rewritten as:

(2) $$\alpha_{T}=\frac{R_{t}-R_{i}}{R_{i}(\Delta T)}$$

### 2.2. Theory of Micro Humidity Sensor

The three main classes of humidity sensors are ceramic, electrolyte-based and polymer-based. Polymer-based sensors are either of the capacitance type or of the resistance type. Although the measurement range of the polymer-based sensor is not as large as that of the ceramic-based sensor, it is simply fabricated, low-cost, and highly accurate because of the high degree of polymer polymerization. It is useful for developing rapid IC processes. The polymer must have high resistance and a low dielectric constant. As the amount of vapor that is absorbed by the polymer increases, the dielectric constant increases and the increase in the capacitance can be derived using Equation (3):

(3) $$C=\varepsilon_{0}\varepsilon (\mathrm{RH})\frac{A}{d}$$

where *C* is capacitance (F); *ε*_0_ is the dielectric constant of a vacuum; *ε* is the dielectric constant of the environment; *A* is cross-sectional area of the electrode (m^2^), and *d* is distance between the two electrodes (m).

**Figure 2.**
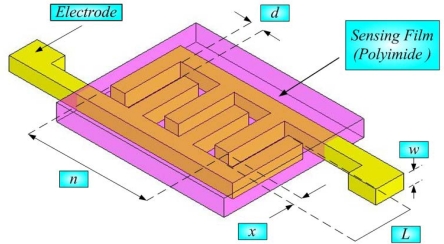
Structure of micro humidity sensor.

Figure 2 displays the structure of the micro humidity sensor, the sensitivity of which is given by Equation (4):

(4) $$\alpha_{H}=\frac{\Delta C}{\Delta \%\mathrm{RH}}$$

where *α_H_* is the sensitivity of the humidity sensor (*C*/%*RH*) [8].

## 3. Fabrication of Flexible Micro Sensors

The frame of the LED is a very important conductor of heat in an LED chip, so an insulating medium is installed between the frame of the LED and the LED chip to increase thermal resistance. The authors’ earlier study involved the development of a flexible micro temperature sensor that could be installed between an LED chip and the frame. The design in the present work is based on that earlier work [9]. In this investigation, micro sensors were fabricated on a stainless steel foil substrate (SS-304 40 μm-thick), and aluminum nitride (AlN) was adopted as an insulation layer, as it has excellent insulating and high thermally conductive properties. As is well known, A1N sputtering yields a film that has pinholes and so is not electrically isolating. To solve this problem, all AlN films that were prepared under the various conditions in this investigation were of satisfactory quality and had a smooth surface, although they did contain some pinhole defects [10]. After the electrical conductivity was tested, their isolating property was confirmed.

**Figure 3.**
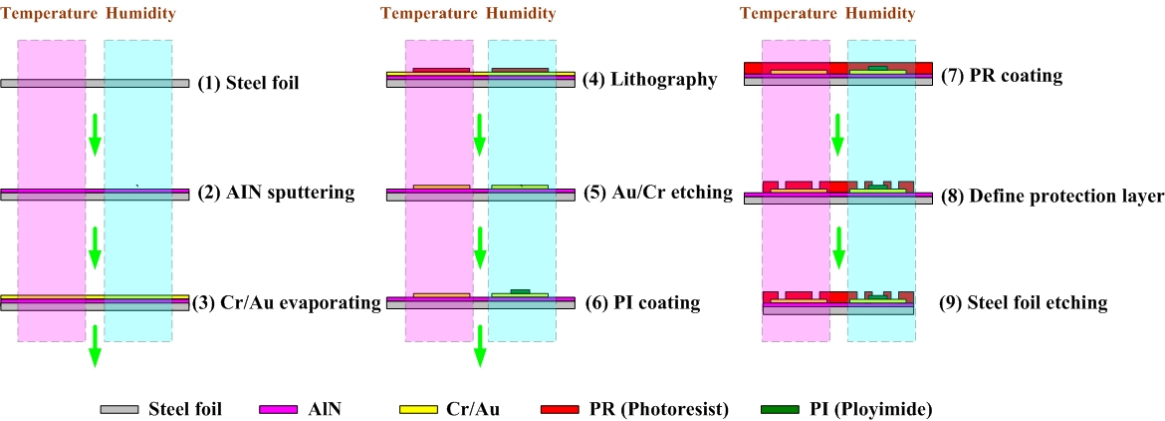
Fabrication of flexible micro sensors.

Figure 3 displays the process for fabricating flexible micro sensors. First, sulfuric acid and hydrogen peroxide were utilized to clean stainless steel foil; AlN was then sputtered (1 μm) as the bottom insulation layer. An E-beam evaporator was then applied to evaporate Cr (200 Å) as an adhesive layer between the AlN and Au, and evaporated Au (2,000 Å) was deposited as the sensing layer. The photoresist (PR) was then spin-coated (3 μm) and the outline of the micro sensors was defined lithographically by wet etching. Polyimide was spin-coated (3 μm) on a humidity sensor as a sensing film. The photoresist (PR) was spin-coated (3 μm) again as a protective layer. Finally, stainless steel foil was etched using aqua regia. Table 1 presents the fabrication process parameters.

## 4. Results and Discussion

**Figure 4.**
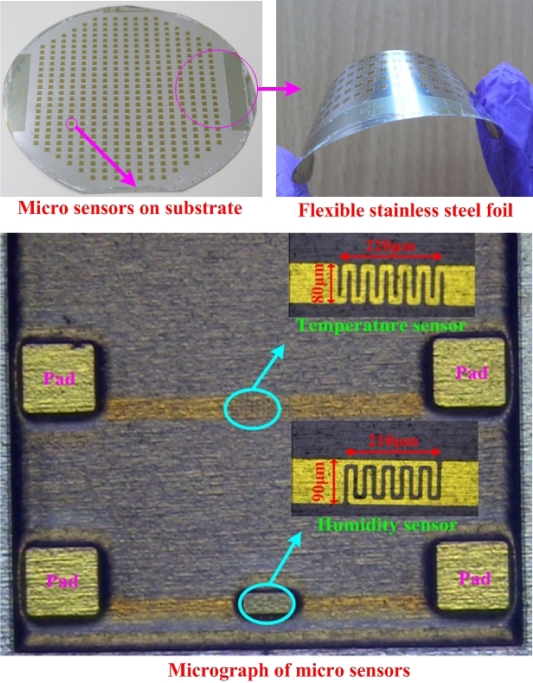
Final flexible micro sensors.

Figure 4 displays an optical microscopic photograph of micro sensors. The sensing area of the micro temperature sensor was 17,600 μm^2^ and the sensing area of the micro humidity sensor was 18,900 μm^2^. Flexible micro temperature and humidity sensors were fabricated using MEMS. Such micro sensors have the advantages of (1) small size, (2) mass-producability, (3) multi-functionality, and (4) flexible but precise measurement positions.

**Figure 5.**
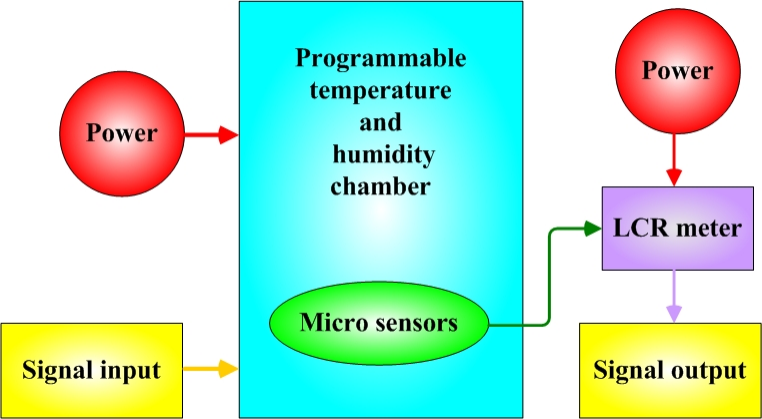
Calibration system of micro sensors.

**Figure 6.**
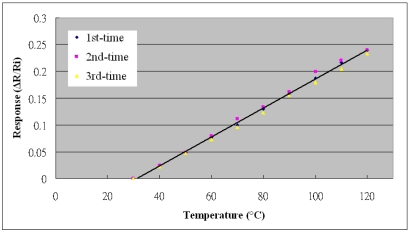
Calibration curve of micro temperature sensor.

**Figure 7.**
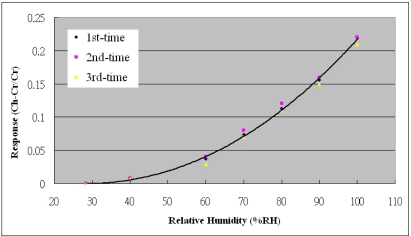
Calibration curve of micro humidity sensor.

Figure 5 presents the calibration system of micro sensors. The micro sensors were placed in a programmable temperature and humidity chamber (Hungta HT-8045 A). The resistance and capacitance of the micro temperature and humidity sensors were measured using an LCR meter. According to the micro temperature and humidity sensors, the temperature and relative humidity varied from 30 to 120 °C and from 28 to 98%RH, respectively. Figure 6 plots the calibration curve of the micro temperature sensor; the result demonstrates that the temperature was almost linearly related to resistance. We have test for three times and the calibration curve is still linear. Figure 7 plots the calibration curve of the micro humidity sensor, which indicates that the capacitance varies as the cube of the humidity. We have test for three times and the calibration curve is cubic equation. The sensitivities of the temperature and humidity sensors were 0.06 ± 0.005 Ω/°C and 0.033 pF/%RH, with accuracies of less than 0.3 °C and 3%RH. The response range is from 1.7 to 2.3 seconds as determined by the Hungta HT-8045A programmable temperature and humidity chamber.

**Figure 8.**
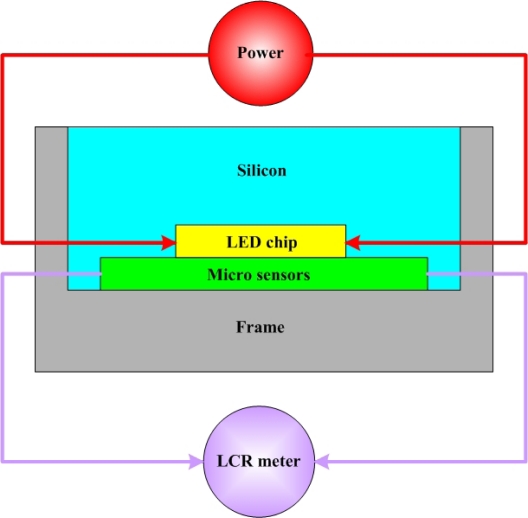
Illustration of micro sensors in LED.

**Figure 9.**
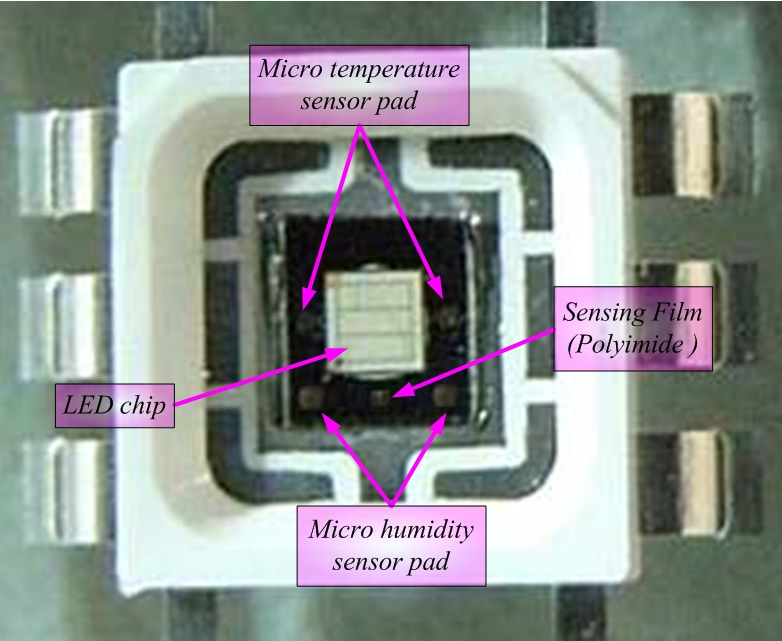
High-power LED with micro sensors.

In Figure 8, the micro sensors are set between an LED chip and a frame, and an input current of 350 mA is passed through the LED, causing it to glow. After temperature and humidity of the LED stabilized, the resistance and capacitance of the micro sensors were measured by an LCR meter, and the results compared to the calibration curve of the micro sensors, to determine their temperature and humidity. The micro sensors require a protective layer, so an insulation layer was deposited on the temperature sensor. However, this insulation layer was very thin, and the thermal gradient was negligible. Figure 9 displays an LED with micro sensors.

The junction temperature of the LED can be determined from the voltage based on the thermal resistance measurement theory. A CREE® EZ1000 chip and input currents ranging from 0 to 350 mA were used. Data obtained by thermal resistance measurement differed from those obtained using a micro temperature sensor. The data experimented is shown in Table 2. The temperature obtained using the micro temperature sensor was 1.52 °C lower than that obtained by thermal resistance measurement at 120 mA, and 5.45 °C lower at 350 mA. Additionally, the variation increased with an increasing temperature.

In the humidity portion, the capacitance that was measured by the humidity sensor was 21.98 pF both before and after packaging. This result suggests that the LED in this study was well-packaged.

## 5. Conclusions

In this study, flexible micro temperature and humidity sensors that could be set between an LED chip and an LED frame was developed, and a 40 μm-thick stainless steel foil was used to conduct heat between the LED chip and the frame. Flexible micro temperature and humidity sensors were fabricated using MEMS. These sensors have the advantages of: (1) small size, (2) high sensitivity, (3) mass-producability, (4) multi-functionality, and (5) flexible but precise measurement positions. A future study will adopt flexible micro temperature and humidity sensors to evaluate the operating parameters of LEDs and thus could improve their design and performance.

## Figures and Tables

**Table 1.** Fabrication of process parameters.

**STEP**	**RECIPE**
(1)	**Steel foil:** stainless steel foil substrate (SS-304 40 μm thick) H_2_SO_4_ + H_2_O_2_ (cleaning 10 mins)
(2)	**AlN sputtering:** Ar (8SCCM), N_2_ (2SCCM), pressure (0.32 Pa), substrate temperature (120 °C), power (170 W)
(3)	**Cr / Au evaporating:** substrate temperature (100 °C), background pressure (7 × 10^−7^Torr), Cr thickness (200 Å), Au thickness (2,000 Å)
(4)	**Lithography:** spin-coated photoresist→ step1:500 rpm, 5s; step2:3,000 rpm, 30 s. soft bake→ 90 °C, 120 s. exposure→ 5 mW/cm^2^. development→ MF319:DI water = 1:1, hard bake→ 90 °C, 300 s
(5)	**Au / Cr etching:** Au etching (KI + I_2_,7 mins), Cr etching (Cr-7, 3 mins)
(6)	**PI coating:** spin-coated polyimide→ step1:500 rpm, 5s; step2:3,000 rpm, 30s. soft bake→ 90 °C, 120 s. exposure→ 5 mW/cm^2^. development→ (HTR D-2):(RER 600) = 1:2, hard bake→ 90 °C, 300 s
(7)	**PR coating:** spin-coated photoresist→ step1:500 rpm, 5 s; step2:3,000 rpm, 30 s. soft bake→ 90 °C, 120 s.
(8)	**Define protection layer:** exposure→ 5m W/cm^2^. development→ MF319:DI water = 1:1, hard bake→ 90 °C, 300 s
(9)	**Steel foil etching:** aqua regia (50 °C)

**Table 2.** Variation in measurements made using micro temperature sensor and thermal resistance measurement technology.

**Operating current (mA)**	**Micro temperature sensor**	**Thermal resistance measurement**
	**Temperature (°C)**	**Temperature (°C)**
0	25.46	25.3
60	36.14	35.1
120	45.32	43.8
180	56.20	53.1
240	66.02	62.5
300	76.06	71.6
350	85.45	80.0
